# Asymptomatic carotid artery stenosis and retinal nerve fiber layer thickness. A community-based, observational study

**DOI:** 10.1371/journal.pone.0177277

**Published:** 2017-05-11

**Authors:** Dandan Wang, Yang Li, Yong Zhou, Cheng Jin, Qi Zhao, Anxin Wang, Shouling Wu, Wen Bin Wei, Xingquan Zhao, Jost B. Jonas

**Affiliations:** 1 Department of Neurology, Beijing Tiantan Hospital, Capital Medical University, Beijing, China; 2 China National Clinical Research Center for Neurological Diseases, Beijing, China; 3 Center of Stroke, Beijing Institute for Brain Disorders, Beijing, China; 4 Beijing Key Laboratory of Translational Medicine for Cerebrovascular Disease, Beijing, China; 5 Beijing Tongren Eye Center, Beijing Key Laboratory of Ophthalmology and Visual Science, Beijing Tongren Hospital, Capital Medical University, Beijing, China; 6 Department of Cardiology, Kailuan Hospital, Tangshan, China; 7 Beijing Institute of Ophthalmology, Beijing Tongren Eye Center, Beijing, Key Laboratory of Ophthalmology and Visual Science, Beijing Tongren Hospital, Capital Medical University, Beijing, China; 8 Department of Ophthalmology, Medical Faculty Mannheim of the Ruprecht- Karls-University, Heidelberg, Germany; Charite Universitatsmedizin Berlin, GERMANY

## Abstract

**Purpose:**

To examine whether an abnormally thin retinal nerve fiber layer (RNFL) is associated with cerebrovascular insufficiency.

**Design:**

Community-based study.

**Methods:**

The Asymptomatic Polyvascular Abnormalities in Community Study included Chinese aged 40+ years and without histories of cerebrovascular incidents or coronary heart disease. Using transcranial Doppler and carotid duplex ultrasound examination, we assessed presence and degree of an intracranial arterial stenosis (ICAS) and extracranial carotid arterial stenosis (ECAS) and we measured the RNFL thickness by spectral-domain optical coherence tomography.

**Results:**

The study included 3,376 participants with a mean age of 54.3±10.3 years. Thinner RNFL was significantly correlated with a higher prevalence of ECAS (*P* = 0.035; standardized regression coefficient beta:-0.04; non-standardized regression coefficient B:-0.99; 95% confidence intervals(CI):-1.90,-0.07), after adjusting for age (*P*<0.001;beta:-0.25;B:-0.26;95%CI:-0.30,-0.22), gender (*P* = 0.001;beta:-0.07;B:-1.36;95%CI:-2.14,-0.58) and blood concentration of low-density lipoproteins (*P* = 0.03;beta:0.04;B:0.52;95%CI:0.05,0.98). In a reverse manner, prevalence of ECAS was associated with a thinner RNFL thickness (*P* = 0.007; odds ratio (OR):0.99; 95%CI:0.98,0.99) after adjusting for older age (*P*<0.001;OR:1.06;95%CI:10.05,10.7), higher prevalence of ICAS (*P* = 0.01;OR:1.34;95%CI:1.07,1.69) and higher prevalence of carotid artery plaques (*P*<0.001;OR:9.18;95%CI:6.93,12.2), and higher blood concentration of total cholesterol (*P* = 0.03;OR:1.12;95%CI:1.01,1.23). In univariate analysis, an increasing degree of ECAS was significantly correlated with a thinner RNFL.

**Conclusions:**

Higher prevalence and degree of ECAS were correlated with thinner RNFL and vice versa. Patients with abnormally thin RNFL without ocular disease may undergo carotid artery examination to detect asymptomatic carotid artery stenosis. Examination of the RNFL is useful for the diagnosis of cerebrovascular disease.

## Introduction

Extracranial carotid artery stenosis (ECAS) and intracranial carotid artery stenosis (ICAS) are one of the main risk factors for ischemic and embolic events in the brain. Cerebral stroke is one of the most common causes for years of life lost (YLL) as shown in the Global Burden of Disease Study 2013 [1,2]. Since treatment of vascular risk factors, antiplatelet therapy and surgical procedures such as carotid endarterectomy, carotid angioplasty and stenting are effective in preventing ischemic cerebrovascular events in patients with symptomatic moderate-grade and high-grade carotid artery stenoses and in some patients with an asymptomatic carotid artery stenosis, detection of a carotid artery stenosis is important, in particular in neurologically asymptomatic patients [3–6]. This raises the question which non-neurological signs could suggest the presence of a carotid artery stenosis.

Since the retina belongs to the end-stream region of the internal carotid artery and since the retinal nerve fiber layer (RNFL) as the inner retinal layer is non-invasively assessable upon ophthalmoscopy and upon refined imaging techniques, we conducted this study to examine whether a thinning of the RNFL is correlated with a neurologically asymptomatic carotid artery stenosis. The hypothesis was that a carotid artery stenosis, also a clinically asymptomatic one, could cause a small ischemic infarct in the RNFL, resulting in a RNFL defect detectable by ophthalmoscopy or by another imaging technique. The examination of the RNFL as extracranial part of the brain by ophthalmoscopy or by spectral-domain optical coherence tomography (OCT) has the advantage of its non-invasiveness and the high spatial resolution of about 10 μm. such a resolution is unsurpassable by any sophisticated neuro-radiological imaging technique of the brain. An association between an abnormal appearance of the RNFL and cerebral small vessel disease and stroke as symptomatic sequels of a carotid artery stenosis has already been reported in other recent investigations [7,8]. The results of our study would further explore the role the examination of the RNFL may play for the assessment of neurologically asymptomatic patients at risk for carotid artery stenosis and cerebral stroke.

## Methods

The Asymptomatic Polyvascular Abnormalities Community study (APAC) is a community-based, observational study to investigate the epidemiology of asymptomatic polyvascular abnormalities and cerebrovascular events and their risk factors in Chinese adults [9]. The Ethics Committee of the Kailuan General Hospital, the Beijing Tongren Hospital and the Beijing Tiantan Hospital approved the study design. All study participants gave their informed written consent. The study cohort was a subgroup of the Kailuan study population which consisted of 101,510 employees and retirees (81,110 men) of the Kailuan Company in Tangshan 135 km East of Beijing. Applying a stratified random sampling method by age and gender based on the data of the Chinese National Census from 2010, we collected a sample of 7000 individuals with an age of 40+ years from the Kailuan study population. A total of 5,852 subjects agreed to participate in the APAC study and 5,816 people eventually completed the baseline examination. A total of 376 individuals were excluded because they did not meet the inclusion criteria (no history of stroke, transient ischemic attack, and coronary disease; and absence of neurologic deficits typically for stroke). The study thus eventually included 5,440 participants at the baseline of the study in 2011. Out of these subjects, 3,376 participants underwent examination of the RNFL at the follow-up examination which took place in 2015. The detail study design and inclusion and exclusion criteria have been descripted in our previous published protocol [9].

For the assessment of the carotid arteries, we performed a transcranial Doppler and carotid duplex ultrasound examination. An ICAS was diagnosed according to the peak blood flow velocity [10]. A stenosis was defined by a peak systolic flow velocity of >140 cm/s for the middle cerebral artery, of >120cm/s for the anterior cerebral artery, >100 cm/s for the posterior cerebral artery and vertebra-basilar artery, and >120 cm/s for the siphon of the internal carotid artery. An ECAS was considered a stenosis of equal to or more than 50% of the extracranial common carotid artery or extracranial internal carotid artery. The severity of the stenosis was graded based on the recommendations made by the Society of Radiologists in Ultrasound Consensus Conference, as <50%, 50–69%, >69% and occlusion [10]. Carotid artery plaques defined as a focal structure either encroaching into the arterial lumen of at least 0.5 mm or 50% of the surrounding intima-media thickness value, or demonstrating a thickness of 1.5 mm from the intima-lumen interface to the media adventitia interface.

Each study participant underwent an interview performed by trained investigators on the basis of a standardized questionnaire (####Questionnaire in Chinese,” and “Questionnaire Translated into English###############). Smoking was defined as at least one cigarette per day for more than a year. Alcohol consumption was considered an intake of at least 80 g of liquor a day for more than 1 year. Smoking or drinking cessation was considered only if it lasted for at least 1 year. Body weight and body height were measured, and the body mass index (BMI) was calculated as body weight (kg) divided by the square of height (m^2^) [11]. Arterial hypertension was defined as self- reported history of hypertension, taking antihypertensive medication or by a systolic blood pressure of ≥140 mm Hg or a diastolic blood pressure of ≥90 mm Hg. Diabetes mellitus was defined by a self-reported history, current treatment with insulin or oral hypoglycemic agents, or a fasting blood glucose concentration ≥7.0mmol/l. Dyslipidemia was defined as a self-reported history, current use of cholesterol lowering medicine, or a total cholesterol level ≥6.22 mmol/l or triglyceride ≥2.26mmol/l or low density lipoprotein ≥4.14mmol/l [12].

Spectral-domain OCT images were taken from the optic nerve head, macula and adjacent retina (iVue SD-OCT, Optovue Inc., Fremont, California, USA). The iVue SD-OCT used a super luminescent diode scan with a center wavelength of 840 ± 10 nm to provide high resolution images. A 6 x 6 mm^2^ raster scan was centered on the optic disc and macula. All OCT scans were obtained through undilated pupils. Quality assurance checks were performed. Images with failures of the segmentation of the RNFL, motion artifacts, poor focusing, a scan score index <40 and images not centered on the optic disc were excluded from the assessment. Two experienced examiners scanned all study participants. Measurements of both eyes of each study participant were taken in a sitting position. For further analysis, we used the mean RNFL thickness from each participant [9].

The statistical analysis was carried out using the SPSS software (version 22.0; IBM-SPSS, Chicago, IL, USA). In a first step, we assessed the distribution of the main parameters by calculating their means, medians and standard deviations. In a second step, we performed a linear regression analysis with RNFL thickness as dependent continuous variable and general parameters as independent parameters. In a third step, we carried out a multivariate linear regression analysis with RNFL thickness as dependent continuous variable and including those variables into the list of independent variables which were significantly associated with RNFL thickness in the univariate analysis. As a corollary, we performed in a fourth step a binary regression analysis with the presence of carotid artery stenosis as dependent variable and RNFL thickness and other variables as independent variables. This analysis was carried out first in a univariate analysis, followed by a multivariate analysis. We calculated the standardized regression coefficient beta and the non-standardized regression coefficient B, odds ratios (OR) and 95% confidence intervals. A *P*-value of less than 0.05 was considered to be statistically significant.

## Results

The study included 3376 participants (1918 (56.8%) men) with a mean age of 54.25 ± 10.28 years (median: 52.3 years; range: 41–89 years) (#########Datafile in SPSS###############). Mean body height was 166 ± 7 cm (median: 166 cm; range: 141–196 cm), mean body weight was 69.2 ± 11.2 kg (median: 69 kg; range: 40–160 kg), and mean body mass index was 25.0 ± 3.3 kg/m^2^ (median: 24.7 kg/m^2^; range: 16–60 kg/m^2^). Systolic blood pressure was 131.9 ± 18.6 mmHg (median: 130 mmHg; range: 80–210 mmHg) and diastolic blood pressure was 82.3 ± 11.7 mm Hg (median: 81.3 mmHg; range: 49–144 mmHg). Mean blood concentration of glucose 5.7 ± 1.7 mmol/L, high-density lipoproteins 1.4 ± 0.5 mmol/L, low-density lipoproteins 2.5 ± 0.8 mmol/L, total cholesterol 5.2 ± 1.0 mmol/L, triglycerides 16.6 ± 1.3 mmol/L, uric acid 298 ± 88 μmol/L, and C-reactive protein was 2.2 ± 3.2 mg/L. Diabetes mellitus was known for 490 (14.5%) study participants. The level of education was primary school or less for 318 (9.4%) individuals, junior high school for 1492 (44.2%) subjects, and high school or higher for 1565 (46.4%) individuals. An intracranial carotid artery stenosis was detected for 699 (20.7%) study participants and an extracranial carotid artery stenosis was detected in 1013 (30.0%) study participants

In univariate linear regression analysis, thinner RNFL thickness was significantly associated with older age (*P*<0.001), male gender (*P*<0.001), higher systolic blood pressure (*P* = 0.001) and higher prevalence of arterial hypertension (*P*<0.001), while body mass index, smoking, alcohol consumption, diastolic blood pressure, blood concentration of glucose, triglycerides, low-density lipoproteins, high-density lipoproteins and C-reactive protein and prevalence of dyslipidemia were not significantly associated with the RNFL thickness (Table 1).

**Table 1:** Univariate analysis of associations between retinal nerve fiber layer (RNFL) thickness and other variables, and baseline characteristics of individuals in the asymptomatic polyvascular abnormalities in community study, stratified into quartile groups of RNFL thickness.

	*P*-Value*	25% RNFL Thickness	50% RNFL Thickness	75% RNFL Thickness	100% RNFL Thickness	*P*-Value
Age (Years)	<0.001	57.0 (47.6, 69.9)	52.5 (46.4, 58.9)	51.6 (46.0, 57.0)	50.0 (45.3, 55.2)	<0.001
Male / Female Gender	<0.001	547 (64.7)	484 (57.4)	469 (55.6)	418 (49.5)	<0.001
Body Mass Index (kg(m^2^)	0.16	24.7 (22.4, 27.0)	24.69 (22.8, 26.9)	24.8 (22.7, 27.0)	24.7 (22.7, 27.0)	0.64
Smoking	0.26	262 (31.0)	281 (33.3)	276 (32.7)	275 (32.6)	0.77
Alcohol Consumption	0.54	265 (31.4)	260 (30.8)	263 (31.2)	244 (28.9)	0.68
Arterial Hypertension, Prevalence (%)	<0.001	546 (64.6)	474 (56.2)	455 (53.9)	419 (49.6)	<0.001
Systolic Blood Pressure (mm Hg)	0.001	132.7 (120.7, 148.0)	130.2 (120.0, 143.3)	129.3 (118.0, 140.7)	129.3 (119.3, 141.3)	<0.001
Diastolic Blood Pressure (mm Hg)	0.63	81.3 (75.0, 90.0)	81.3 (75.0, 90.0)	80.7 (73.3, 89.3)	81.3 (73.3, 90.0)	0.15
Diabetes, Prevalence (%)	0.10	131 (15.5)	125 (14.8)	113 (13.4)	121 (14.3)	0.66
Dyslipidemia, Prevalence (%)	0.07	366 (43.3%)	385 (45.7)	347 (41.1)	340 (40.3)	0.11
Blood Concentration of:						
Glucose (mmol/L)	0.98	5.30 (4.90, 5.83)	5.30 (4.86, 5.96)	5.31 (4.88, 5.83)	5.30 (4.82, 5.91)	0.99
Cholesterol (mmol/L)	0.04	5.03 (4.50, 5.82)	5.11 (4.55, 5.77)	5.08 (4.45, 5.70)	5.09 (4.45, 5.73)	0.70
Triglycerides (mmol/L)	0.20	1.26 (0.91, 1.86)	1.23 (0.90, 1.81)	1.21 (0.92, 1.88)	1.21 (0.87, 1.93)	0.76
Low-Density Lipoproteins (mmol/L)	0.12	2.38 (1.78, 3.00)	2.45 (1.91, 3.08)	2.48 (1.89, 3.03)	2.51 (2.00, 3.03)	0.08
High-Density Lipoproteins (mmol/L)	0.69	1.33 (1.13, 1.59)	1.38 (1.13, 1.60)	1.38 (1.17, 1.63)	1.38 (1.13, 1.60)	0.30
C-Reactive Protein (mmol/L)	0.70	1.30 (0.64, 2.56)	1.32 (0.69, 2.40)	1.32 (0.65, 2.40)	1.24 (0.63, 2.33)	0.57

*P*-Value*: Results of the linear regression analysis of RNFL thickness as linear variable with variables

We then conducted a multivariable linear regression analysis with RNFL thickness as dependent variable and as independent variables all those variables which were significantly associated with RNFL thickness in the univariate. Out of the list of independent variables, we dropped step by step arterial hypertension (*P* = 0.96) and systolic blood pressure (*P* = 0.75) due to a lack of statistical significance. In the final model thinner RNFL was significantly correlated with a higher prevalence of ECAS after adjusting older age (*P*<0.001; beta -0.25; B: -0.26; 95%CI: -0.30, -0.22), male gender (*P* = 0.001; beta -0.07; B: -1.36; 95%CI: -2.14, -0.58) and lower blood concentration of low-density lipoproteins (*P* = 0.03; beta 0.04; B: 0.52; 95%CI: 0.05, 0.98) (Table 2).

**Table 2:** Associations between retinal nerve fiber layer thickness (μm) (Linear multivariate regression analysis) and other variables in the asymptomatic polyvascular abnormalities in community study.

Variable	*P*-Value	Standardized Regression Coefficient beta	Non- Standardized Regression Coefficient B	95% Confidence Interval of B
Prevalence of Carotid Artery Stenosis	0.035	-0.04	-0.99	-1.90, -0.07
Age (Years)	<0.001	-0.25	-0.26	-0.30, -0.22
Gender (Men / Women)	0.001	-0.07	-1.36	-2.14, -0.58
Blood Concentration of Low-density Lipoproteins (mmol/L)	0.03	0.04	0.52	0.05, 0.98

In a reverse manner, in a binary regression analysis, the prevalence of ECAS was significantly associated with a thinner RNFL thickness (*P* = 0.007) after adjusting for older age (*P*<0.001), higher prevalence of ICAS (*P* = 0.01) and higher prevalence of plaques in the carotid arteries (*P*<0.001), and higher blood concentration of total cholesterol (*P* = 0.03) (Table 3).

**Table 3:** Associations between the prevalence of extracranial carotid artery stenosis and retinal nerve fiber layer thickness (μm) (Binary multivariate regression analysis) in the asymptomatic polyvascular abnormalities in community study.

Variable	*P*-Value	Odds Ratio	95% Confidence Interval of Odds Ratio
Retinal Nerve Fiber Layer Thickness (μm)	0.007	0.99	0.98, 0.99
Age (Years)	<0.001	1.06	1.05, 1.07
Prevalence of Intracranial Carotid Artery Stenosis	0.01	1.34	1.07, 1.69
Prevalence of Plaques in the Carotid Arteries	<0.001	9.18	6.93, 12.2
Blood Concentration of Total Cholesterol	0.03	1.12	1.01, 1.23

## Discussion

In our study population, patients with an asymptomatic stenosis of the carotid arteries had a significantly thinner RNFL than individuals without carotid artery stenosis, in univariate analysis, and in multivariate analysis with adjusting for variables such as older age and higher prevalence of smoking. In a reverse manner, thinner RNFL was associated with prevalence and degree of ECAS in univariate analysis, and in multivariate analysis after adjusting for older age, male gender and lower blood concentration of low-density lipoproteins.

The results of our study concur with the findings obtained in two previous investigations on patients with symptomatic carotid artery stenosis and on patients with risk factors for a cerebrovascular insufficiency and carotid artery stenosis. In a study by Wang and colleagues on 154 patients with acute ischemic stroke and 2890 subjects from the population-based Beijing Eye Study as control group, acute stroke was significantly associated with a thinning of the RNFL (*P*<0.001; odds OR: 6.23) after adjusting for older age, male sex, arterial hypertension, diabetes mellitus, and higher concentration of the C-reactive protein [8]. In a similar manner, previous stroke was correlated with a higher prevalence of thinning of the RNFL in multivariate analysis. In a reverse manner, the presence of a thinning of the RNFL was related with cerebral stroke (*P*<0.001; OR: 3.54) after adjusting for age, sex, and prevalence of diabetes mellitus. In an investigation by Kim and associates on 4395 Korean individuals undergoing health check-up examination, higher prevalence of thinning of the RNFL was significantly correlated with cerebral small vessel diseases as detected by magnetic resonance imaging (OR: 1.58; 95% CI: 1.17–2.12) after adjusting for arterial hypertension, male gender, and older age [7]. The findings of our study extend the observations made in the previous studies in that thinning of the RNFL is associated also with clinically asymptomatic stenosis of the carotid arteries.

The findings obtained in our study are in agreement with the results of other studies which showed an association between cerebrovascular diseases and other changes in the retina, i.e., retinal microvascular abnormalities such as localized and generalized arteriolar thinning, arterio-venous nicking and a lower arteriolar / venular diameter ratio [13–16]. Investigations also showed correlations between the retinal microvasculature and arterial hypertension as one of the main risk factors of cerebrovascular disorders including carotid artery stenosis [17–20]. Correspondingly, the Atherosclerosis Risk in Communities study revealed that retinal microvascular abnormalities were associated with MRI-defined cerebral infarcts after adjusting for relevant stroke risk factors [21]. The Blue Mountains Eye Study reported that retinal emboli were associated with the occurrence of systemic vascular diseases and with some stroke risk factors such as hypertension and smoke [22]. Although retinal microvascular abnormalities are associated with a thinning of the RNFL and although thinning of the RNFL is correlated with risk factors for cerebrovascular disorders including carotid artery stenosis, none of these studies showed associations between RNFL thinning and carotid artery stenosis [23].

Our study is in line with other investigations which also applied spectral-domain OCT to measure the RNFL in patients with other neurological diseases such as Parkinson´s disease, schizophrenia or Alzheimer's disease [24–26]. One may infer that spectral-domain OCT may be added to the retinal imaging diagnostic tests in patients with some neurological disorders. It may hold true also for patients with an asymptomatic carotid artery stenosis which is one of the most important risk factors for eventual ischemic cerebral stroke. Cerebral stroke is worldwide one of the most common causes for years of life lost (YLLs), in particular in China, where it cerebrovascular disease is the most frequent cause of YLLs [1]. Since reduction of risk factors for carotid artery stenosis including change of diet and lifestyle and reduction of arterial blood pressure is therapeutically effective, early detection of carotid artery stenosis in the asymptomatic state is clinically warranted. Assessment of the RNFL may be one of the steps to achieve that goal.

Potential limitations of our study should be discussed. First, our study population was a randomly selected subgroup out of the larger population of the Kailuan Study, which, despite its large sample size was not fully representative for the Chinese population. Our study sample however was drawn from the Kailuan study population by using a stratified random sampling method based on the Chinese National Census from 2010. In that context, one may also discuss the "healthy worker effect", including the "healthy hire" and "healthy survivor" effect, that is present in many occupational cohort studies. It may be associated with a confounding effect. It was however not the goal of our study to examine the prevalence of carotid artery stenosis in the general Chinese population but to assess potential associations between carotid artery stenosis and RNFL abnormalities. Second, our study was a cross-sectional investigation the study design of which did not allow drawing longitudinal conclusions on a causal relationship between RNFL thickness and atherosclerotic changes of the carotid arteries. The study design could have been improved if RNFL thickness measurements had been obtained at baseline of the whole study, and a future study may assess changes in RNFL thickness parallel to changes in the status of the carotid arteries. Third, the study population included Han Chinese, so that it has remained unclear whether the findings of our study can be transferred onto other ethnic groups. Fourth, ICAS was evaluated by transcranial Doppler sonography and ECAS was evaluated by duplex sonography what may be more precise than transcranial Doppler sonography to detect atherosclerotic changes. Also, we did not apply magnetic resonance angiography or other refined methods for the detection of arterial stenosis. It is however, almost not feasible to use magnetic resonance angiography in a community-based study on several thousand asymptomatic individuals. Fifth, the odds ratio of the association between the occurrence of an ECAS and RNFL thickness was 0.99 in the multivariate analysis, a value which was at the margin of a diagnostic benefit. Correspondingly, thinner RNFL thickness was associated with the presence of an ECASs with a relatively low standardized regression coefficient beta of -0.04 (Table 2), again suggesting a relatively impact of the association. Despite the statistical significance of the association one may therefore keep in mind, that the importance of the association between ECAS and thinner RNFL thickness was marginal from a practical point of view.

In conclusion, a higher prevalence and a more marked degree of ECAS were correlated with a thinner RNFL. It supports the notion that patients with an abnormally thin RNFL without an ocular disease explain the thinning of the RNFL may undergo carotid artery examination to detect asymptomatic carotid artery stenosis.

## Supporting information

S1 File Strobe checklist. (DOC)

S2 File Datafile in SPSS. (SAV)

S3 File Questionnaire in Chinese. (PDF)

S4 File Questionnaire translated into English. (DOCX)
