# Supplementary material for: Asymptomatic carotid artery stenosis and retinal nerve fiber layer thickness. A community-based, observational study
Source: PLoS One. 2017 May 11;12(5):e0177277. doi: 10.1371/journal.pone.0177277 (PMC5426666; doi:10.1371/journal.pone.0177277)
Supplement: S3 File — (PDF) [file pone.0177277.s003.pdf]

开滦集团公司员工
健康随访表

（重点人群）

体 检 人 编 号 97335

体 检 人 姓 名 李晓明

体检日期 2012-9-5

开滦医疗集团编印

2012—2013

填写说明

1. 填写记录请使用蓝黑色钢笔或签字笔清楚填写。
2. 体检表填写务必准确、清晰，不得随意涂改，错误之处纠正时需用红色钢笔或签字笔在错误处居中划一道横线，并签署修改者姓名及修改时间，原改处应能清晰可见。不要掩盖填入的原始数据，不要用橡皮擦、用改正液遮盖或划许多道线。
3. 举例：~~3.24~~ 8.2 李某某 07/04/28

   ~~☒ ×××~~　　☒ ×××　李某某 07/04/28

   ~~⊗ ×××~~　　⊗ ×××　李某某 07/04/28

4. 根据职工本人的具体情况，按要求填写登记表的每一项。“O”为单项选择，“□”为多项选择。在“□”内或“O”处划“×”表示选择此项（如：☒或）。在“⊗”内填写有关的文字说明，要求字迹工整，应用规范的医学术语，不得应用简写或英文缩写。
5. 缺失或不可得的信息，请在相应的横线上面填写“NA”。
6. 所有表格上的日期都以“年/月/日”的形式表示，包括职工本人的出生日期。如果不知道具体日期，请用“NA”表示，以“年/月/ NA”的形式填入日期，请尽可能填入完整的阳历日期。所有时间均采用24 小时计时制，如下午 5 点应填写为“17:00”。

体检编号□□□□□□

一、 个人健康信息

1. 基本信息

1.1 联系电话（固定）：3563520　　联系电话（移动）：13832564800

1.2 体重(kg)：70kg

1.3 腰围(cm)：88　　臀围(cm)：107　　腹围(cm)：91

1.4 血压(mmHg)　第一次：____/____　第二次____/____　第三次____/____

2. 高血压病史

2.1 上次体检（2010 年-2011 年）后，是否被医生诊断为**高血压病**

☑ 1-否（跳转到 3）　　O 2-是

2.2 首次诊断时间：______年____月

2.3 目前是否服用降压药：O 1-否　　O 2-是

2.4 目前正在服用药物：□ 1-利尿剂　□ 2-β 受体阻滞剂　□ 3-钙拮抗剂
□ 4-血管紧张素转换酶抑制剂
□ 5-血管紧张素 II 受体拮抗剂　□ 6-α 受体阻断剂
□ 7-复合制剂　□8-中药制剂及其他

3. 糖尿病史

3.1 上次体检（2010 年-2011 年）后，是否被医生诊断为**糖尿病**

☑ 1-否(跳转到 4)　　O 2-是

3.2 首次诊断时间 ：______年____月

3.3 糖尿病类型：O 1-1 型　　O 2-2 型　　O 99-不详

3.4 是否服用降糖药：O 1-否　　O 2-是

3.5 服用药物种类：□ 1-磺脲类　□ 2-双胍类
□3-葡萄糖苷酶抑制剂　□ 4-噻唑烷二酮类
□ 5-非胰岛素促分泌剂
□ 6-胰岛素制剂　□ 7-中药及其他

4. 高脂血症病史

4.1 上次体检（2010 年-2011 年）后，是否被医生诊断为**高脂血症**

☑ 1-否(跳转到 5)　　O 2-是

4.2 首次诊断时间：______年____月

4.3 是否服用调脂药：O 1-否　　O 2-是

4.4 调脂药物种类：
□1-他汀类 □2-贝特类 □3-胆酸螯合树脂类 □4-烟酸及其衍生物 □5-中药制剂及其他
若选择他汀类，请选择具体的药物名称：__________

5. 自上次体检以来，是否在医院被诊断为以下疾病：

5.1 □ **房颤/房扑**　　诊断时间：______年____月

5.2 □ **心力衰竭**　　诊断时间：______年____月

5.3 □ **心肌梗死**　　诊断时间：______年____月

5.3.1 诊断医院：__________

5.4 □脑卒中，请选择卒中类型：

O1-缺血性卒中 诊断时间： ________年____月
O2-脑出血 诊断时间： ________年____月
O3-蛛网膜下腔出血 诊断时间： ________年____月
O4-短暂性脑缺血发作（TIA）诊断时间： ________年____月
O5-其他 诊断时间： ________年____月

5.4.1 诊断医院：________________

5.5 □肿瘤，请选择肿瘤类型：

O1-有，乳腺癌 诊断时间： ________年____月
O2-有，肺癌 诊断时间： ________年____月
O3-有，胃癌 诊断时间： ________年____月
O4-有，胰腺癌 诊断时间： ________年____月
O5-其他 诊断时间： ________年____月

5.5.1 诊断医院：________________

6. **月经生育史（仅限女性填写）**

孕产史：

6.1 共怀孕：__1__次

其中 分娩：__1__次，自然流产（<28 周）：__0__次，人流：__0__次，药流：__0__次；

6.2 初孕年龄：__26__岁，初产年龄：__26__岁；末孕年龄：__26__岁，末产年龄：__26__岁；

6.3 分娩方式： ☑1-阴道顺产 □2-阴道助娩 □ 3-剖宫产

6.4 是否长期服用口服避孕药？ ☑1-否 O2-是

6.4.1 如“是”，使用的年限：________年

月经史：

6.5 您的月经初潮是：__17__岁（周岁）末次月经日期：2012年 2月 日

6.6 您既往月经状况

6.6.1 20 岁前是否规律？☑1-规律 O 2-基本规律 O 3-完全无规律

6.6.2 20 岁—35 岁间是否规律？☑1-规律 O 2-基本规律 O 3-完全无规律

绝经相关情况调查：

6.7 是否做过妇科手术？ ☑1-否 O 2-是

6.7.1 如“是”，手术范围是

O 1-切除子宫，保留双侧卵巢 O 4-保留子宫，切除一侧卵巢
O 2-切除子宫，同时切除一侧卵巢 O 5-保留子宫，切除双侧卵巢
O 3-切除子宫，同时切除双侧卵巢 O 6-其他__________

6.7.2 手术时是否已经绝经？ O 1-否 O 2-是

6.8 您是否已经绝经？ O 1-否 ☑2-是 O 3-不清楚（已切除子宫，手术时仍有月经） 如“是”，绝经年龄：__54__岁 （本次月经结束已满一年）

6.9 若没有绝经，您目前的月经状况属于

O 1-与以前完全一样（跳转到 6.14）
O 2-育龄晚期（月经有轻微变化，包括月经量和周期的变化）
O 3-绝经过渡期早期（月经周期与年轻时比，变化超过 7 天，且末次月经在 2 个月以内）
O 4-绝经过渡期晚期（末次月经在 2 个月以前，1 年以内）

6.10 **绝经前月经开始变化的年龄：** ____53____ 岁

6.11 **绝经前的月经变化首先是**

6.11.1 月经量变化　○ 0-无变化　○ 1-变少　○ 2-变多　○ 3-时多时少

6.11.2 月经周期长度变化　○ 0-无变化　○ 1-变长　○ 2-变短　○ 3-时长时短

6.11.3 月经量变化和周期长度变化同时发生　○ 0-无　○ 1-有

6.12 **绝经前月经变化过程**

○ 1-月经稀发，量少，至停止　○ 2-月经稀发，量多，至停止

○ 3-月经频发，量多，至停止　○ 4-月经频发，量少，至停止

○ 5-月经周期完全无规律，时长时短　○ 6-月经突然停止

○ 99-其它

6.13 **绝经过程中的月经变化是否对您造成了困扰？**　○ 1-否 ○ 2-是

6.13.1 若选是，是否因此看医生？　○ 1-否 ○ 2-是

6.14 **绝经过程中是否有更年期相关症状？**　○ 1-否 ○ 2-有

6.14.1　**如"有"，症状出现时间与月经变化之间的关系：**

○ 1-先出现月经变化，后出现症状

○ 2-先出现症状，后出现月经变化

○ 3-两者同时出现

6.14.2　如"有"，为缓解更年期症状，是否用过以下治疗方法？

6.14.2.1 中医中药　○ 1-否　○ 2-是

6.14.2.2 植物雌激素（大豆异黄酮）○ 1-否　○ 2-是

如"是"，具体名称 __________

6.14.2.3 保健品（如：蜂胶、太太口服液）　○ 1-否　○ 2-是

如"是"，具体名称__________

6.15 是否使用过激素补充疗法？ ○ 1-否　○ 2-是

6.15.1　如"是"，开始使用时间 ：__________年_____月

6.15.2　目前是否使用？ ○ 1-否　○ 2-是

6.15.3　累计使用年限：__________年

6.15.4　**使用药物种类：**

○ 1-雌激素类

口服雌激素　□倍美力 □补佳乐 □维尼安 □其它__________

经皮雌激素　□松奇　□其它____________________

经阴道雌激素　□更宝芬 □欧维婷 □其它____________

○ 2-孕激素类　□安宫黄体酮　□天然黄体酮胶丸/胶囊

□达芙通　□其它____________________

○ 3-替勃龙　□利维爱　□紫竹爱维

○ 4-复方制剂　□克龄蒙　□芬吗通　□安今益

□倍美罗　□其它____________________

6.16 **您目前是否有下列不适症状？**

体检编号□□□□□□

* 严重程度以0—10之间的数字表示，请选择相应的程度（"0"代表无症状，"10"代表症状极严重，按从轻到重选择"0－10"之间的恰当反映严重程度的数字）
* 影响最大的症状：从11个症状中限选一项，在相应的栏目中划"×"

	严重程度<br>无症状 轻度 ——→ 重	持续时间（年）	影响最大症状
1 潮热出汗	0 1 2 3 4 5 6 7 8 9 10	2	O
2 心脏不适	0 1 2 3 4 5 6 7 8 9 10		O
3 睡眠问题	0 1 2 3 4 5 6 7 8 9 10		O
4 情绪抑郁	0 1 2 3 4 5 6 7 8 9 10		O
5 易怒	0 1 2 3 4 5 6 7 8 9 10		O
6 焦虑	0 1 2 3 4 5 6 7 8 9 10		O
7 行为／记忆受损	0 1 2 3 4 5 6 7 8 9 10		O
8 性生活问题	0 1 2 3 4 5 6 7 8 9 10		O
9 泌尿系统问题	0 1 2 3 4 5 6 7 8 9 10		O
10 阴道干涩	0 1 2 3 4 5 6 7 8 9 10		O
11 关节肌肉症状	0 1 2 3 4 5 6 7 8 9 10		O

二、 饮食、生活习惯

1. **吸烟史**

1.1 是否吸烟

O1-现在吸 O2-从不吸烟 O3-曾经吸，现已戒烟>12个月 O4-曾经吸，现已戒，戒烟<12个月

1.2 （若现在吸烟）每日平均吸烟支数

O1-1—10支 O2-11—20支 O3-21—30支

O4-31—40支 O5-41—50支 O6-51—60支 O7-61支及以上

1.3 是否被动吸烟（家庭或工作环境中经常有人吸烟）

O0-无 O1-有

1.3.1 如有，被动吸烟的年限________年

2. **饮酒史**

2.1 饮酒频率

O0- 不饮酒
O1-<1个标准饮酒量/日
O2-<2个标准饮酒量/日
O3-2~4个标准饮酒量/日
O4-≥5个标准饮酒量/日
O5-饮酒，但剂量不详
O6-已戒酒
O99-不详（不知是否饮酒）

（1个标准饮酒量相当于120ml[2.5两]葡萄酒、360ml[1听]啤酒或45ml[1两左右]白酒）

饮酒的度数

3. **体育锻炼及膳食（自上次体检到现在）**

4. **是否经常饮茶** O1-从不饮 O2-每月少于 1 次 O3-每月 1—3 次 O4-每周 1—3 次 O5-每周最少 4 次 O6-每天 1-2 次 O7-每天 3 次及以上

4.1 饮茶的主要种类： O1-红茶（比如普洱茶） O2-绿茶 O3- 花茶 （比如茉莉花茶、菊花茶） O4-其他（比如大麦茶）

5. **食盐习惯：** O1-喜淡（<6 克盐/日） O2-一般（6-12 克盐/日） O3-喜咸（>12 克盐/日）

6. 在 1976 年唐山大地震时，您是否在唐山震区？

O 1-不在 O 2-在

6.1 如果在，您是否受伤？

O 1-否 O 2-是，头颈部受伤 O 3-是，躯干部受伤，O 4-是，其他部位受伤；O 5-是，多个部位受伤

7. 您是否有家人因 76 年大地震去世？

O 1-没有 O 2-有，1 人 O 3-有，2 人 O 4-有，3 人或更多 O 5-是，不记得人数

8. 在大地震后的 1－2 年间，您是否居住在唐山？

O 0- 不住在唐山 O 1-是，平均每年超过 9 个月 O 2- 是，平均每年 6—9 个月 O 3-是，平均每年 3—5 个月 O 4- 是，平均每年少于 3 个月

三、 颈部血管超声（无碳复写）

		IMT mm	狭窄率
颈总动脉	左	0.9	O✓1-正常（未报狭窄） O 2-< 50%<br>O 3- 50～ 69% O 4- 70%～接近闭塞<br>O 5-接近闭塞 O 6-完全闭塞
	右	0.9	O✓1-正常（未报狭窄） O 2-< 50%<br>O 3- 50～ 69% O 4- 70%～接近闭塞<br>O 5-接近闭塞 O 6-完全闭塞
颈内动脉	左	0.8	O✓1-正常（未报狭窄） O 2-< 50%<br>O 3- 50～ 69% O 4- 70%～接近闭塞<br>O 5-接近闭塞 O 6-完全闭塞
	右	0.8	O✓1-正常（未报狭窄） O 2-< 50%<br>O 3- 50～ 69% O 4- 70%～接近闭塞<br>O 5-接近闭塞 O 6-完全闭塞
颈外动脉	左	0.8	O✓1-正常（未报狭窄） O 2-< 50%<br>O 3- 50～ 69% O 4- 70%～接近闭塞<br>O 5-接近闭塞 O 6-完全闭塞
	右	0.8	O✓1-正常（未报狭窄） O 2-< 50%<br>O 3- 50～ 69% O 4- 70%～接近闭塞<br>O 5-接近闭塞 O 6-完全闭塞
锁骨下起始段	左	0.9	O✓1-正常（未报狭窄） O2-狭窄 <50%<br>O3-狭窄 >50%
	右	1.0	O✓1-正常（未报狭窄） O2-狭窄 <50%<br>O3-狭窄 >50%
椎动脉	左		O✓1-正常（未报狭窄） O 2-< 50%<br>O 3- 50～ 69% O 4- 70%～接近闭塞<br>O 5-完全闭塞
	右		O✓1-正常（未报狭窄） O 2-< 50%<br>O 3- 50～ 69% O 4- 70%～接近闭塞<br>O 5-完全闭塞

斑块参数	血管	有无斑块	位置	斑块数目	回声性质
斑块	右侧颈总	O✓1-无斑块（未报斑块）<br>O2-有斑块	O1-分叉处<br>O2-干部<br>O3-全程	O1-单发<br>O2-多发	O1-等回声 O2-强回声<br>O3-低回声 O4-混合回声
	右侧颈内	O✓1-无斑块（未报斑块）<br>O2-有斑块	O1-起始处<br>O2-分叉—颈内	O1-单发<br>O2-多发	O1-等回声 O2-强回声<br>O3-低回声 O4-混合回声
	右侧锁骨下	O✓1-无斑块（未报斑块）<br>O2-有斑块	O1-起始处	O1-单发<br>O2-多发	O1-等回声 O2-强回声<br>O3-低回声 O4-混合回声
	左侧颈总	O✓1-无斑块（未报斑块）<br>O2-有斑块	O1-分叉处<br>O2-干部<br>O3-全程	O1-单发<br>O2-多发	O1-等回声 O2-强回声<br>O3-低回声 O4-混合回声
	左侧颈内	O✓1-无斑块（未报斑块）<br>O2-有斑块	O1-起始处<br>O2-分叉—颈内	O1-单发<br>O2 多发	O1-等回声 O2-强回声<br>O3-低回声 O4-混合回声

友情提示：此结果为体检结果，如有异常，请专科门诊复查并确诊

体检编号□□□□□□

四、TCD 检查（无碳复写）

血管名称	左侧	右侧
大脑中动脉	增快<br>O 1-正常 O 2-轻度 O 3-中度<br>O 4-重度 O 5-闭塞 O 6-未探及	O 1-正常 O 2-轻度 O 3-中度<br>O 4-重度 O 5-闭塞 O 6-未探及
颈内动脉末端	O 1-正常 O 2-狭窄 O 3-未探及	O 1-正常 O 2-狭窄 O 3-未探及
大脑前动脉	O 1-正常 O 2-狭窄 O 3-未探及	O 1-正常 O 2-狭窄 O 3-未探及
大脑后动脉	O 1-正常 O 2-狭窄 O 3-未探及	O 1-正常 O 2-狭窄 O 3-未探及
颈内动脉虹吸部	增快<br>O 1-正常 O 2-狭窄 O 3-未探及	O 1-正常 O 2-狭窄 O 3-未探及
椎动脉	O 1-正常 O 2-狭窄 O 3-未探及	O 1-正常 O 2-狭窄 O 3-未探及
基底动脉	O 1-正常 O 2-狭窄 O 3-未探及	

友情提示：此结果为体检结果，如有异常，请专科门诊复查并确诊

体检编号□□□□□□

五、 国际体力活动调查表

本调查表中的问题是针对您在过去7天中进行各种体力活动的时间，包括工作，交通行程，家务劳动，以及闲暇时间（体育锻炼、娱乐活动）中的各项体力活动。您的回答很重要。即使您认为自己不是个爱活动的人，也请您对每项问题作出回答。 感谢您的配合

在下列问题中，
● 重体力活动是指需要您花费大力气完成，呼吸较平常明显增强的活动。
● 中等强度体力活动是指需要您花费中等力气完成，呼吸较平常稍微增强的活动。

在回答下面的问题时，请只考虑那些每次至少十分种的体力活动。

1a 在过去7天中，您有几天进行重体力活动，例如搬（举）重物，跑步，游泳，踢足球，打篮球，打网球，跳绳，跳迪斯科，健身房内跳健身操等？（只计算那些每次至少*10*分钟的活动）。

O 1-每周______天

O 2-没有（0）（跳至问题*2a*）

1b 在这几天中，您每天进行这些重体力活动的时间

O 1-平均为每天 ______ 小时 ______ 分钟

2a 在过去7天中，您有几天进行中等强度体力活动，如搬（举）轻物，骑自行车，打太极拳，十八法，关节操，扇子舞，木兰拳，乒乓球，羽毛球，交谊舞等？不要包括步行。（只计算那些每次至少*10*分钟的活动）。

O 1-每周______天

O 2-没有（0）（跳至问题*3a*）

2b 在这几天中，您每天进行这些中等强度体力活动的时间为

O 1-平均每天 ______小时 ______ 分钟

2c 请选择具体的活动类型：

O 1-骑自行车 O 2-打太极拳 O 3-乒乓球，羽毛球 O 4-交谊舞，扇子舞等

3a 在过去7天中，您有几天每次步行至少10分钟？ 这里的步行包括您工作时和在家中的步行，交通行程的步行以及为了锻炼身体进行的步行。

O 1-每周__7__天

O 2-没有（0）（跳至问题*4a*）

3b 在这几天中，您每天步行的时间为

O 1-平均每天 __8__小时 ______ 分钟

4 最后的问题是关于您处于静坐的时间，包括您在工作单位和家中，坐在办公桌前，电脑前，坐着或躺着看电视，拜访朋友，看书，乘车等的时间。

O 1-在过去7天中，您每天处于静坐的时间大约为平均每天__8__小时______ 分钟

六、 眼科检查

1. 您曾经被确诊为青光眼吗？

O1 是　　　O2 否（跳转到二）　O3 不清楚

2 若是，请注明诊断医院：__________

3 若是，诊断____年

4 若是，请选择治疗手段（可多选）：

□1）手术　□2）药物　□3）打激光　□4）治疗了，但不清楚　□5）未治疗

2. 您是否做过白内障手术？

O1 是　　O2 否

如果是，单眼还是双眼？

O1 单眼　　O2 双眼

0259 - 0266

2 手术是哪年做的？______

O左眼___年；O右眼___年

3. 视力

1）裸眼视力：右眼 1.2　　左眼 1.2　　99 不适用

2）生活视力：右眼______　　左眼______　　99 不适用

4. 验光

右眼 +0.50/-0.25　93　99 不适用

左眼 +0.50/　　99 不适用

眼科生活质量专门化量表（VF-14）	
**友情提示：好眼最佳生活视力≤0.3 的受访者请继续回答如下问题：**	
1. 不费力；2. 有一点费力；3. 困难；4. 非常困难；5. 因为视力不好，已经不做了；6. 因为其他原因不做这类事情了	
1）阅读小号的字体，如药的说明书、黄页电话薄、食物标签等	（ ）
2）阅读普通书报或书	（ ）
3）阅读大号字体的书或报，或者手机/电话上显示的号码	（ ）

4) 认出近处的人	( )
5) 看台阶、楼梯等	( )
6) 看交通标志、路标或商店招牌	( )

体检编号□□□□□□

七、 认知功能调查表

Mini-Mental State Examination（MMSE）简易精神状态检查

项目	不正确	正确
**定向力**		
1. 今天是这个月的几号?	0 ☐	1 ☑
2. 今年是哪一年?	0 ☐	1 ☑
3. 现在是几月份?	0 ☐	1 ☑
4. 今天是星期几?	0 ☐	1 ☑
5. 现在是什么季节?	0 ☐	1 ☑
6. 这家医院的名字是什么?	0 ☐	1 ☑
7. 我们现在是在第几层楼?	0 ☐	1 ☑
8. 我们现在在哪个城市（城镇）?	0 ☐	1 ☑
9. 我们现在在哪个省（或州、或地区、或县、或岛屿）?	0 ☐	1 ☑
10. 我们现在在哪个国家?	0 ☐	1 ☑
**即刻记忆**		
现在我要说三样东西的名称，在我讲完之后，请您重复说一遍，请您记住这三样东西，因为等一下要再问您的：（以第一次回答记分）		
11. 回答出“皮球”	0 ☐	1 ☑
12. 回答出“国旗”	0 ☐	1 ☑
13. 回答说“树木”	0 ☐	1 ☑
**注意力与计算能力**		
100 减 7，连续减 5 次（若错了，但下一个回答是对的，得 1 分）		
14. 回答出“93”	0 ☐	1 ☑
15. 回答出“86”	0 ☐	1 ☑
16. 回答出“79”	0 ☐	1 ☑
17. 回答出“72”	0 ☐	1 ☑
18. 回答出“65”	0 ☐	1 ☑
**回忆能力**		
现在请您告诉我，刚才我要您记住的三样东西是什么?		
19. 回答出“皮球”	0 ☐	1 ☑
20. 回答出“国旗”	0 ☐	1 ☑
21. 回答说“树木”	0 ☐	1 ☑
**语言能力**		
访问员拿出手表，并问“请问这是什么?”然后再拿出一支铅笔，并询问同样问题。		
22. 回答出“手表”	0 ☐	1 ☑
23. 回答出“铅笔”	0 ☐	1 ☑
**重复**		
24. 请您跟我说：“四十四只石狮子。”	0 ☐	1 ☑
三步命令：给患者一张白纸并告诉他/她，“用您的右手拿起纸，对折并把纸放在		

地板上”。		
25. 患者用右手拿起纸？	0 □	1 ☑
26. 患者将纸对折？	0 □	1 ☑
27. 患者把纸放在地板上？	0 □	1 ☑
**阅读：访问员把写有“闭上您的眼睛”大字的卡片交给患者，请患者照着这张卡片所写的去做。**		
28. 患者闭上了眼睛？	0 □	1 ☑
**书写：请患者写一句完整的、有意义的句子（必须有主语、动词、并有意义）记下句子。**		
29. 患者写了一个句子？	0 □	1 ☑
**复制**		
30. 请您按样子画图	0 □	1 ☑

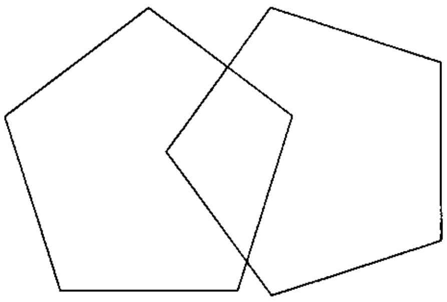

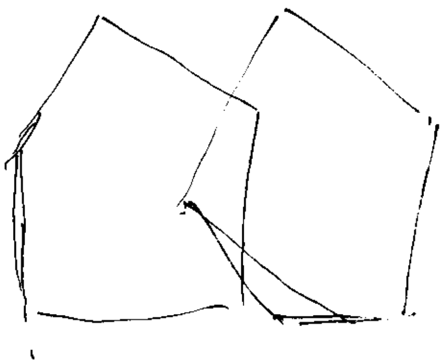

文化程度： □1=文盲 □2=小学 □3=中学及以上

得分： [illegible]分

八、 认知功能附加表

Montreal Cognitive Assessment (MoCA) Beijing Version
蒙特利尔认知评估北京版

出生日期：
教育水平：
性　别：
姓名：
检查日期：

视空间与执行功能

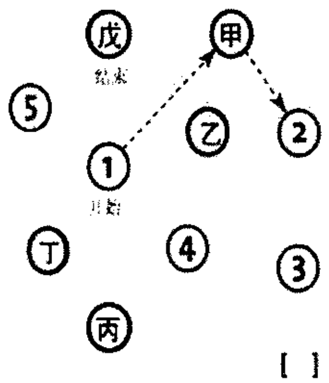

[ ]

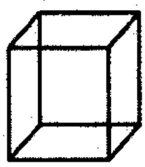

复制立方体 [ ]

画钟表（11点过10分）（3分）

[ ] 轮廓　[ ] 数字　[ ] 指针　__/5

命名

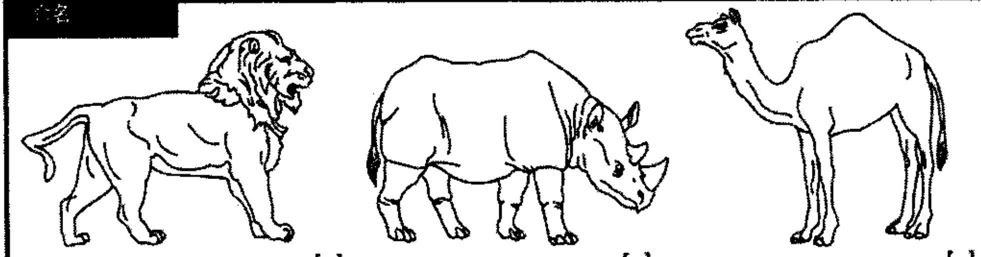

[ ]　[ ]　[ ]　__/3

记忆

读出下列词语，而后由患者重复上述过程重复2次，5分钟后回忆

	面孔	天鹅绒	教堂	菊花	红色
第一次					
第二次					

不计分

注意

读出下列数字，请患者重复（每秒1个）　顺背 [ ] 2 1 8 5 4　倒背 [ ] 7 4 2　__/2

读出下列数字，每当数字1出现时，患者必须用手敲打一下桌面，错误数大于或等于2个不给分
[ ] 5 2 1 3 9 4 1 1 8 0 6 2 1 5 1 9 4 5 1 1 1 4 1 9 0 5 1 1 2　__/1

100连续减7　[ ] 93　[ ] 86　[ ] 79　[ ] 72　[ ] 65　__/3
4-5个正确给3分，2-3个正确给2分，1个正确给1分，全部错误为0分

语言

重复：我只知道今天张亮是来帮过忙的人 [ ]
狗在房间的时候，猫总是躲在沙发下面 [ ]　__/2

流畅性：在1分钟内尽可能多的说出动物的名字　[ ] ____ (N ≥ 11名称)　__/1

抽象

词语相似性：如香蕉-桔子=水果　[ ] 火车-自行车　[ ] 手表-尺子　__/2

延迟回忆

回忆时不能提示	面孔	天鹅绒	教堂	菊花	红色	仅根据非提示回忆计分
	[ ]	[ ]	[ ]	[ ]	[ ]	
选项 分类提示						
多选提示						

__/5

定向

[ ] 日期　[ ] 月份　[ ] 年代　[ ] 星期几　[ ] 地点　[ ] 城市　__/6

总分　__/30

© Z.Nasreddine MD Version November 7, 2004
Beijing version 26 August, 2006 translated by Wei Wang & Hengge Xie
www.mocatest.org

ID:97335　　测量日期:2012年09月05日　10:00:59

姓名: LLIXIAOMING

身高 :160cm　　体重 : 70kg　　性别: 女性　　年龄: 53岁

BMI : 27.3kg/m²　　症状:　　用药:

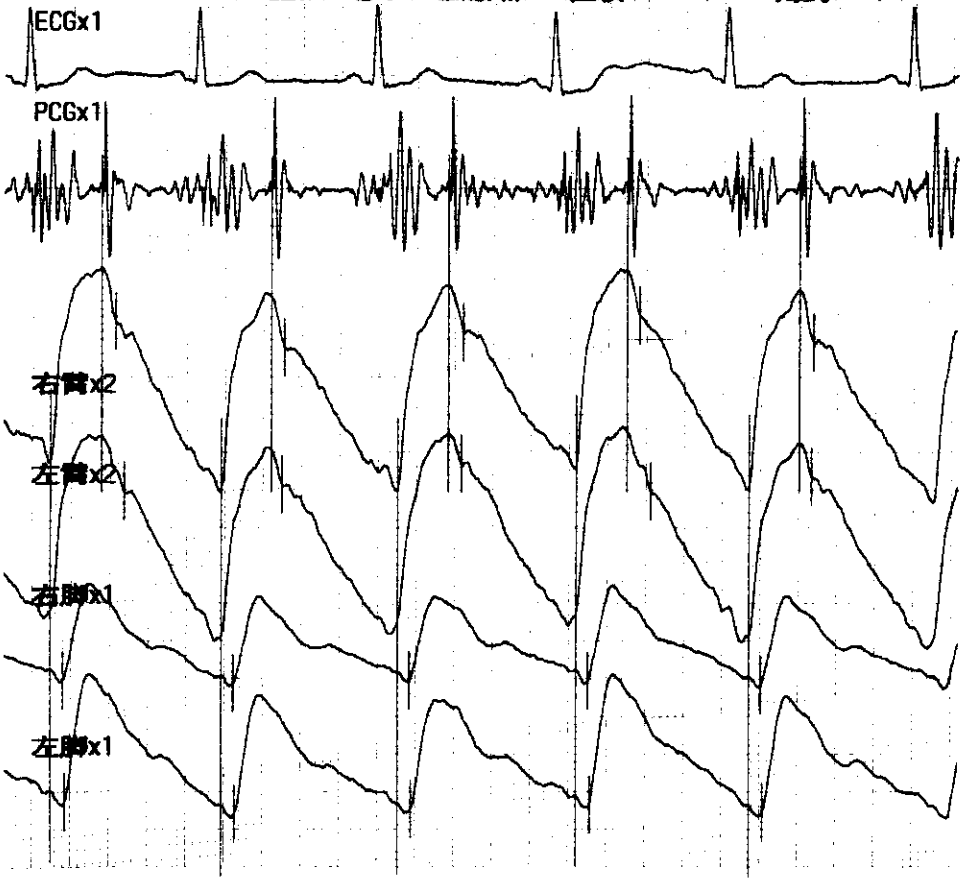

L	=	L1	+	L2	+	L3
123cm		63cm		33cm		27cm

R-*CAVI*: 8.9

L-*CAVI*: 8.3

右臂血压 : 159/ 91 mmHg

脉压 : 68 mmHg

R-*CAVI*

tb: 73ms tba: 64ms Tha:137ms

L-*CAVI*

tb: 73ms tba: 72ms Tha:145ms

VS-1000(No.99999) CN YMD V13-01　　P50　25mm/s

ID:97335

测量日期:2012年09月05日 10:00:59

姓名: LLIXIAOMING

身高 :160cm 体重 : 70kg 性别: 女性 年龄: 53岁

AF: 484mm BMI : 27.3kg/m² 症状: 用药:

L 123cm = L1 63cm + L2 33cm + L3 27cm

检查目的	检查项目	测量值	标准值	检查结果
动脉的硬化程度	R-CAVI	8.9	~ 9.0	血管年龄超过年龄代。
	L-CAVI	8.3	7.8±0.7	血管年龄与龄年代相同。
动脉的堵塞程度	R-ABI	1.33	0.91~1.40	正常范围。
	L-ABI	1.25		正常范围。

HR: 64(拍/分)

右上臂 [mmHg]
血压(S/D): 159/ 91
脉压: 68 平均:129

左上臂 [mmHg]
血压(S/D): 160/ 90
脉压: 70 平均:129

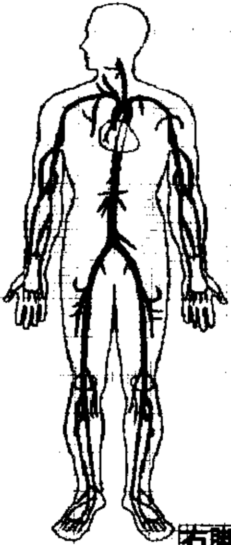

右脚腕 [mmHg]
血压(S/D): 212/ 85
脉压:127 平均:132

左脚腕 [mmHg]
血压(S/D): 199/ 79
脉压:120 平均:142

R-CAVI: 8.9
R-ABI: 1.33

L-CAVI: 8.3
L-ABI: 1.25

右脚 这次为正常范围
左脚 这次为正常范围

VS-1000(No.99999) CN YMO V13-01
