# Supplementary material for: Asymptomatic carotid artery stenosis and retinal nerve fiber layer thickness. A community-based, observational study
Source: PLoS One. 2017 May 11;12(5):e0177277. doi: 10.1371/journal.pone.0177277 (PMC5426666; doi:10.1371/journal.pone.0177277)
Supplement: S4 File — (DOCX) [file pone.0177277.s004.docx]

Section 1 Personal Healthy Information

1. Basic Information
   1. Contact Phone
   2. Weight (kg)
   3. Waist (cm) _____________ Hipline (cm) ______________ Abdominal Girth(cm) _________
   4. Blood Presure (mmHg) First __________ Second _________ Third_______
2. History of Hypertension
   1. Diagnosed as Hypertension since last physical examination
3. No (Turn to 3) 2. Yes
   1. The first diagnosed time ______ Year _____ Month
   2. Anti-hypertensive medicine
4. No 2. Yes
5. History of diabetes
   1. Diagnosed as diabetes since last physical examination
6. No (Turn to 3) 2. Yes
   1. The first diagnosed time ______ Year _____ Month
   2. Type of diabetes
7. Type 1 2. Type 2
   1. Anti-diabetic medicine
8. No 2. Yes
9. History of hyperlipidemia
   1. Diagnosed as hyperlipidemia since last physical examination
10. No (Turn to 3) 2. Yes
    1. The first diagnosed time ______ Year _____ Month
    2. Lipid-lowering medicine
11. No 2. Yes
12. Diagnosed as following diseases since last physical examination

5.1 Atrial fibrillation diagnosed time ______Year ______Month

5.2 Cardiac failure diagnosed time ______Year ______Month

5.3 Myocardial infarction diagnosed time ______Year ______Month

5.3.1 Diagnosed Hospital _______

5.4 Stroke

1. Ischemic stroke diagnosed time ______Year ______Month

2. ICH diagnosed time ______Year ______Month

3. SAH diagnosed time ______Year ______Month

4. TIA diagnosed time ______Year ______Month

5. Others diagnosed time ______Year ______Month

5.4.1 Diagnosed Hospital ______

5.5 Tumors diagnosed time ______Year ______Month

Section 2 Dietary and Lifestyle Habit

1, History of smoking

1.1 Smoking

1. Now 2. Never 3. Former, quit smoking > 12 months 4. Former, quit smoking < 12 months

1.2 If smoking, how many per days

1. 1-10 2. 11-20 3. 21-30 4. 31-40 5. 41-50 6. 51-60 7. >=61

1.3 Passive smoking

0. No 1. Yes

2. History of drinking

2.1 Frequency

0. No drinking 1. <=1 Unit/day 2. <2 Units/Day 3. 2-4 Units/Day 4. >= 5 Units/day 5. Uncertain Unit 6. Quit drinking 99. Unknown

(One Unit = 120 ML wine, 360 ML beer, or 45 ML white spirit)

Section 3 Carotid artery ultrasound

| Location | Left/Right | IMT(mm) | Rate of Stenosis |
| --- | --- | --- | --- |
| CCA | Left  Right |  | 1 2 3 4 5 6  1 2 3 4 5 6 |
| ICA | Left  Right |  | 1 2 3 4 5 6  1 2 3 4 5 6 |
| ECA | Left  Right |  | 1 2 3 4 5 6  1 2 3 4 5 6 |
| SubA | Left  Right |  | 1 2 3 4 5 6  1 2 3 4 5 6 |
| VA | Left  Right |  | 1 2 3 4 5 6  1 2 3 4 5 6 |

1. Normal 2. <50% 3. 50% - 69% 4. 70% - 99% 5. Almost 100% 6. Occlusion

|  | Artery | Plaque | Number | Echo |
| --- | --- | --- | --- | --- |
| PLAQUE | Right CCA | 1. No 2. Yes | 1. Single  2. Multiple | 1. Equal  2. Strong  3. Low  4. Mix |
|  | Right ICA | 1. No 2. Yes | 1. Single  2. Multiple | 1. Equal  2. Strong  3. Low  4. Mix |
|  | Right SubA | 1. No 2. Yes | 1. Single  2. Multiple | 1. Equal  2. Strong  3. Low  4. Mix |
|  | Left CCA | 1. No 2. Yes | 1. Single  2. Multiple | 1. Equal  2. Strong  3. Low  4. Mix |
|  | Left ICA | 1. No 2. Yes | 1. Single  2. Multiple | 1. Equal  2. Strong  3. Low  4. Mix |
|  | Left SubA | 1. No 2. Yes | 1. Single  2. Multiple | 1. Equal  2. Strong  3. Low  4. Mix |

Section 4 TCD

| Artery | Left | Right |
| --- | --- | --- |
| MCA | 1. Normal  2. Mild  3. Moderate  4. Severe  5. Occlusion  6. Not Found | 1. Normal  2. Mild  3. Moderate  4. Severe  5. Occlusion  6. Not Found |
| ICA Terminal | 1. Normal  2. Stenosis  3. Not Found | 1. Normal  2. Stenosis  3. Not Found |
| ACA | 1. Normal  2. Stenosis  3. Not Found | 1. Normal  2. Stenosis  3. Not Found |
| PCA | 1. Normal  2. Stenosis  3. Not Found | 1. Normal  2. Stenosis  3. Not Found |
| ICA siphon | 1. Normal  2. Stenosis  3. Not Found | 1. Normal  2. Stenosis  3. Not Found |
| VA | 1. Normal  2. Stenosis  3. Not Found | 1. Normal  2. Stenosis  3. Not Found |
| BA | 1. Normal  2. Stenosis  3. Not Found | 1. Normal  2. Stenosis  3. Not Found |
